# An agonist of the CXCR4 receptor is therapeutic for the neuroparalysis induced by *Bungarus* snakes envenoming

**DOI:** 10.1002/ctm2.651

**Published:** 2022-01-24

**Authors:** Marco Stazi, Federico Fabris, Kae Yi Tan, Aram Megighian, Alessandro Rubini, Andrea Mattarei, Samuele Negro, Giorgia D'Este, Florigio Lista, Ornella Rossetto, Choo Hock Tan, Cesare Montecucco

**Affiliations:** ^1^ Department of Biomedical Sciences University of Padova Padova Italy; ^2^ Department of Molecular Medicine Faculty of Medicine University of Malaya Kuala Lumpur Malaysia; ^3^ Department of Pharmaceutical and Pharmacological Sciences University of Padova Padova Italy; ^4^ Center of Medical and Veterinary Research of the Ministry of Defense Policlinico Militare Rome Italy; ^5^ Department of Pharmacology Faculty of Medicine University of Malaya Kuala Lumpur Malaysia; ^6^ CNR Institute of Neuroscience Padova Italy

Dear Editor,

Here, we report the therapeutic properties of a small molecule agonist of the CXCR4 receptor on the recovery from the peripheral flaccid neuroparalysis caused by *Bungarus* snakebites. Snake envenoming is a neglected disease that, each year, causes >100 000 deaths in tropical and sub‐tropical areas of the world and leaves >400 000 envenomed patients with permanent disabilities, with associated high social costs (https://www.who.int/news‐room/fact‐sheets/detail/snakebite‐envenoming). This is a disease of poverty believed by experts to be under reported.^1^ Moreover, snake envenoming strikes particularly in lower income and rural parts of the world where advanced hospital care may not be rapidly available to the envenomed patient.

A major number of neuropathological snakebites are produced by snakes of the *Bungarus* genus widely distributed in Asia.^2^ Their venoms induce a descending flaccid paralysis with respiratory failure and autonomic dysfunctions. A large reduction of the function of the neuromuscular junction (NMJ) can be recorded electrophysiologically within few hours from snakebite,^2^ and death may occur by respiratory failure. Mechanically ventilated patients survive because the *Bungarus* neurotoxins do not kill motor neurons but induce a rapid degeneration limited to the axon terminals which is followed by a slow regeneration requiring prolonged hospitalization (3–5 weeks) and eventual recovery.^2^, ^3^ However, hospitalization with mechanical ventilation poses major problems in low‐income countries associated with high costs and risks of in‐hospital complications.

The pathophysiology of *Bungarus* snake envenoming is primarily driven by neurotoxicity caused by protein neurotoxins.^2^, ^3^ The presynaptically acting β‐bungarotoxins (β‐BTX) are phospholipases A_2_ that cleave the ester bond of the fatty acid in the sn‐2 site of the glycerol moiety of phospholipids, causing accumulation of fatty acids and lysophospholipids leading to rapid degeneration of axon terminals within few hours in mice.^4^ This is followed by regrowth of the motor axon terminal with reformation of a functional NMJ. This remarkable process is almost complete within about a week in young mice and can be monitored by imaging the NMJ using presynaptic protein markers and by electrophysiology.

Recently we discovered that the degeneration of the motor axon terminal is accompanied by the expression of the CXCR4 receptor on the neuronal plasma membrane^5^ and that a small molecule CXCR4 agonist, dubbed NUCC‐390,^6^ promotes the recovery of function of the NMJ degenerated by the spider α‐latrotoxin.^7^ Therefore, we investigated the utility of NUCC‐390 in treating neurotoxicity induced by the venoms of three most common Asiatic krait species, that is, *Bungarus caeruleus, Bungarus candidus* and *Bungarus multicinctus*.^8^

Figure 1 and Figure S1 show that the venoms cause a rapid and complete degeneration of the motor axon terminals leaving intact the convoluted post‐synaptic structure of the NMJ. This is accompanied by the surface expression of the CXCR4 receptor (Figure 1 Suppvideo), and the CXCR4 staining is concentrated on nerve axon stumps close to perisynaptic Schwann cells (Figure S2). CXCR4 persists for a long time period, to decline by day 7, when recovery of the NMJ is accomplished. Figure 1 also shows that this phenomenon is due to β‐BTX and not to α‐BTX which is a post‐synaptic neurotoxin present in some of the *Bungarus* venoms. These findings provide the basis for testing the effect of the CXCR4 receptor agonist NUCC‐390 on NMJ anatomical and functional regeneration.

**FIGURE 1:**
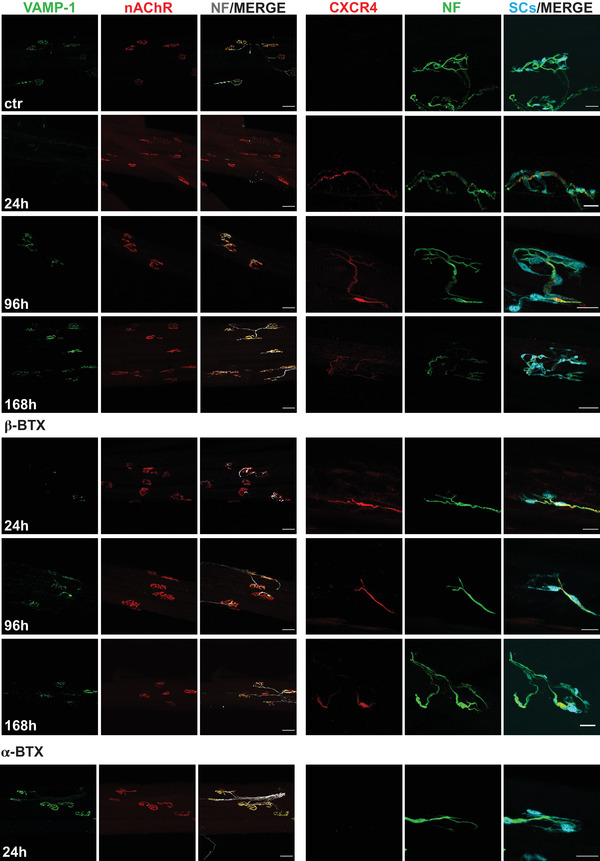
CXCR4 receptor expression on mouse neuronal axons after i.m. injection of *Bungarus caeruleus* venom, β‐ and α‐bungarotoxins. 36 ng/g *B. caeruleus* venom (2nd, 3rd and 4th rows), 1 ng/g of β‐bungarotoxin (5th, 6th and 7th rows) and 1 ng/g of α‐bungarotoxin (bottom row) were injected i.m. in the hind limb of young mice, and the soleus muscle was dissected and stained with antibodies specific for the following antigens. Left set of panels: first column: vesicle‐associated membrane protein isoform‐1 (VAMP‐1), a marker of motor axon terminals; second column: nicotinic acetyl choline receptor (nAChR), a marker of the post‐synaptic muscle membrane, was stained with fluorescent alpha‐bungarotoxin; third column: merging with the staining obtained with an anti‐neurofilaments (chicken polyclonal, Abcam, cat# Ab4680) in grey. The right set of panels shows samples of soleus muscle, poisoned as above, stained with: first column: anti CXCR4 receptor (rabbit monoclonal, Abcam, cat# Ab124824) (red); second column: anti‐neurofilaments (NF, green), third column, merging with Schwann cells expressing GFP (light blue) showing that the CXCR4 staining is concentrated on nerve axon stumps close to the perisynaptic Schwann cells. Symbols of the rows: Samples taken from non‐injected control muscles (ctr), or venom treated muscles after 24 h, 96 h and 168 h from i.m. injection of *Bungarus caeruleus* venom in the hind limb. Notice that the degeneration of the motor axon terminal at the neuromuscular junction with time is complete after 24 h from poisoning as shown by the loss of VAMP‐1 staining. Degeneration is accompanied by the expression of the CXCR4 receptor on the remaining motor axon, and this receptor is still present 4 days after poisoning. A lower magnification was used (scale bar 50 μm) in most panels to provide a view of several neuromuscular junctions in the same field, whilst a higher one (scale bar 20 μm) was used in other panels to better show the expression of the CXCR4 receptor

Functional recovery of the NMJ was quantitatively assessed by electrophysiology performed on single muscle fibres (evoked junctional potential) or on the muscle (compound muscle action potential [CMAP]). Figure 2 shows that NUCC‐390 accelerates the recovery of NMJ function from the neuroparalysis caused by three different *Bungarus* venoms or β‐BTX. Figure 2D shows that the NUCC‐390 effect is mediated by its action on the CXCR4 receptor as it is completely prevented by the very specific antagonist AMD3100. This finding on single muscle fibres is paralleled by the one assessed on the entire muscle by the measurement of CMAP (Figure 3) and by imaging the NMJ using the specific presynaptic marker vesicle‐associated membrane protein isoform‐1 (VAMP‐1) (Figure S3).

**FIGURE 2:**
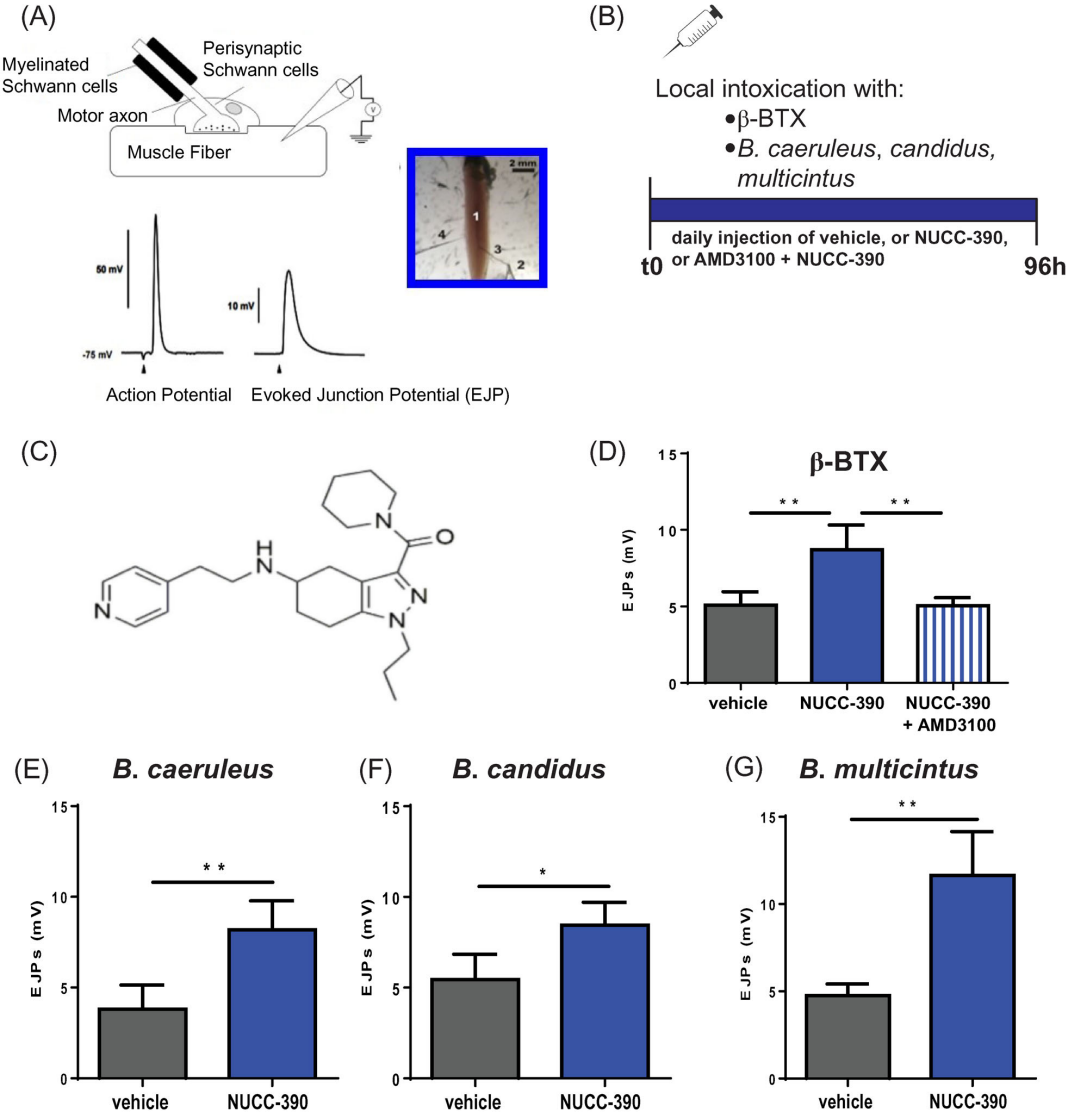
NUCC‐390 promotes the recovery of evoked junction potentials (EJPs) of neuromuscular junctions of single muscle fibres of the mouse soleus muscles injected with the venoms of *Bungarus caeruleus* , or *Bungarus candidus* or *Bungarus multicinctus* or β‐bungarotoxin. (A) Schematic representation of the technique of measurement of the evoked junctional potentials (EJPs) by insertion of the recording electrode inside the muscle fibre. (B) Timeline scheme of the injections of toxin or venoms (quantities as in Figure 1) and of NUCC‐390 administration (3.2 mg/Kg in 20‐μl physiological solution containing .2% gelatine per day) or vehicle only or with additional i.p. injection of 100 μg AMD3100 (in 40‐μl physiological solution containing 0.2% gelatine, prior to NUCC‐390 injection). (C) Chemical formula of CXCR4 agonist dubbed NUCC‐390. (D) EJPs of soleus muscles 96‐h post injection of β‐bungarotoxin in the hind limb of mice daily treated with NUCC‐390 (blue column) or vehicle (grey column) or NUCC‐390 plus the CXCR4 antagonist AMD3100 (striped column). Each bar represents the mean of the EJP amplitude ± SEM from *N* = 5 soleus muscles, number of analysed fibres for each muscle: 12, **p = .0077. (E, F and G) EJPs of soleus muscles 96 h after the injection of the indicated snake venoms in the hind limb of mice treated (blue columns) or not treated (grey columns) daily with NUCC‐390. Each bar represents the mean of the EJP amplitude ± SEM from *N* = 5, number of analysed fibres: 12, (E) **p = .0099, (F) *p = .0193, (G) **p = .0019

**FIGURE 3:**
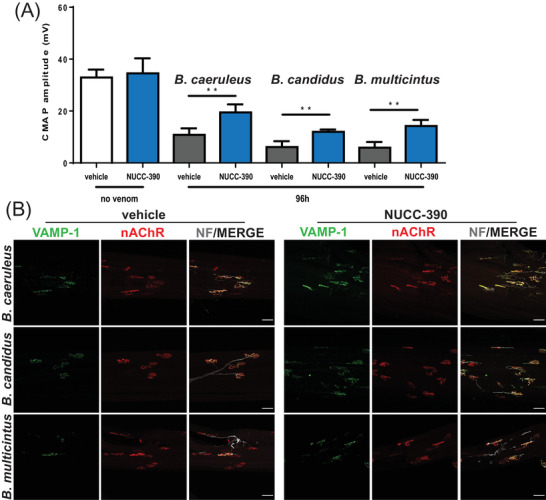
Accelerated recovery of compound muscle action potential from the paralysis caused by *Bungarus* venoms induced by NUCC‐390. (A) Compound muscle action potential (CMAP) values recorded on gastrocnemius muscles 96 h after mock treatment or injection with *B. caeruleus*, *B. candidus*, or *B. multicinctus* venoms, w/o NUCC‐390 daily local administration, using concentration as in Figure 2. The venom affects all the muscles around the site of the injection in mice. Data are expressed as CMAP amplitude (mV) ± SEM. (*B. caeruleus*) **p = .0049, (*B. candidus*) **p = .0028, (*B. multicinctus*) **p = .005. **p < .01. (B) Representative immunostaining of intoxicated neuromuscular junctions (NMJs), performed on the same muscles used for CMAP analysis. Motor neurons axon terminals are identified by vesicle‐associated membrane protein isoform‐1 (VAMP‐1) immunostaining (green), post‐synaptic nicotinic acetyl choline receptors (nAChRs) by fluorescent α‐BTX (red) and the axon by neurofilaments (NF) staining (chicken polyclonal, Abcam, cat# Ab4680) (white). Scale bars: 50 μm

As *Bungarus* envenoming results in deficit of lung ventilation,^2^, ^3^ NUCC‐390 was tested on the recovery of the respiratory function in envenomed mice using a very sensitive and minimally invasive test. A probe connected to a pressure sensor was placed inside the esophagus at the mediastinum level in anesthetized mice. Figure 4 shows the recorded signal characterized by asymmetric peaks with a frequency corresponding to events of lung ventilation. The peak area provides an estimate of the volume of air inspired which results from the activity of respiratory muscles. *Bungarus* venoms decrease both frequency and extent of ventilation with changes in shape and dimension of each peak (Figure 4A). NUCC‐390‐treated animals showed an increase in peak area compared to untreated mice after 24 h, and this effect amplified with time. At 96 h, NUCC‐390‐treated mice showed a complete recovery in lung ventilation, a result reached by control animals only after 1 week. A quantitative analysis shows that, after the initial decline due to envenomation (4‐h point in the graph of panel C of Figure 4), NUCC‐390 accelerates the recovery of ventilation after envenomation (panel B) as compared to controls. This functional recovery is accompanied by the restoration of the structure of diaphragm NMJs as shown in Figure 4D.

**FIGURE 4:**
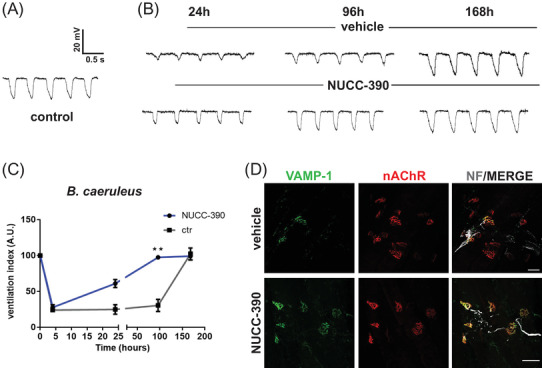
Faster recovery of lung ventilation in mice treated with the *Bungarus caeruleus* venom. A ventilation index was estimated by recording mediastinum pressure variations by placing a plastic feeding tube carrying a pressure sensor placed in the lower third of the esophagus, at the level of mediastinum. (A) Representative traces of mediastinum pressure variations in untreated mouse or (B) in a mouse after 24/96/168 h after injection of *B. caeruleus* venom (36 ng/gr) (upper traces), or in a mouse envenomed with the *B. caeruleus* venom and daily i.p. injected with NUCC‐390, (lower traces of panel B). (C) Quantitative assay performed by measuring the peak area of 20 consecutive peaks of the traces (±SEM) obtained from a group of *N* = 4 mice. (D) Representative immunostaining of intoxicated neuromuscular junction (NMJ), performed on the diaphragm muscle 96 h after the intoxication with *B. caeruleus* not treated (upper panels) or i.p. treated with NUCC‐390 daily (lower panels). Motor neurons axon terminals are identified by vesicle‐associated membrane protein isoform‐1 (VAMP‐1) immunostaining (green), post‐synaptic muscle membrane by nicotinic acetyl choline receptor (nAChR) staining with fluorescent α‐BTX (red). Axon is identified by neurofilaments (NF) staining (chicken polyclonal, Abcam, cat# Ab4680) (white). Scale bars: 50 μm

Taken together, the data presented here qualify NUCC‐390 as a novel potential therapeutic to accelerate nerve structural and functional recovery from the neurodegeneration and paralysis caused by *Bungarus* snake envenoming. The molecule is stable, non‐toxic, of simple chemical synthesis^7^ and does not suffer from the limitations of anti‐venom antisera because its efficacy does not depend on the species specificity and origin of the antiserum and can be administered also even days after snakebite.^9^, ^10^ In fact, NUCC‐390 is complementary to antisera because of its different mechanism of action which leads to a more rapid recovery from a peripheral neuroparalysis, thereby shortening the mechanical ventilation period with a parallel decrease of healthcare costs. This is important particularly in developing countries where most of neuroparalytic snakebites occur. These properties and considerations support the proposal of a rapid translation of NUCC‐390 to trials in envenomed patients.

## CONFLICT OF INTEREST

The authors declare no conflict of interest.

## Supporting information

Figure S1. CXCR4 receptor expression on mouse neuronal axons after i.m. injection of *Bungarus candidus* or *Bungarus multicintus* venoms in mice. 22 ng/g of *B. candidus* venom or 22 ng/g of *B. multicintus* venom was injected in the hind limb of mice, and the soleus muscle was isolated and stained with antibodies specific for the following antigens: first column) VAMP‐1, a marker of motor axon terminals; second column) nicotinic acetyl choline receptor (nAChR), a marker of the post‐synaptic muscle membrane, was stained with fluorescent α‐BTX (α‐BTX, third column) merging with the antibody staining neurofilaments (NF) (chicken polyclonal, Abcam, cat# Ab4680) in grey. The right set of panels displays the staining of samples of soleus muscle, poisoned as above and stained with: first column) anti‐CXCR4 receptor (rabbit monoclonal, Abcam, cat# Ab124824) (red): second column) NF (green); third column) merging with Schwann cells stained in light blue. Symbols of the rows: Samples taken from non‐injected control muscles (ctr); venom treated muscles after 24 h, 96 h and 168 h from i.m. injection of *B. candidus* or *B. multicintus* venoms in the hind limb. Notice that the degeneration of the motor axon terminal at the NMJ is complete after 24 h from poisoning as shown by the loss of VAMP‐1 staining. This is accompained by the expression of the CXCR4 receptor on the residual motor axon; this receptor is still present after 4 days from poisoning. A lower magnification was used for the left panels (scale bar 50 μm) to provide a view of several NMJs in the same field, whilst a higher one (scale bar 20 μm) was used in the right panels to better show the expression of the CXCR4 receptor.

Figure S2. CXCR4 receptor expression on mouse neuronal axons after i.m. injection of *Bungarus caeruleus* venom. (A) Note that 36 ng/g *B. caeruleus* venom were injected i.m. in the hind limb of young mice, and the soleus muscle was dissected 96 h after and stained with antibodies specific for the following antigens: anti‐CXCR4 receptor (rabbit monoclonal, Abcam, cat# Ab124824) (red); anti‐neurofilaments (chicken polyclonal, Abcam, cat# Ab4680) in green, merging with Schwann cells expressing GFP (light blue) showing that the CXCR4 staining is concentrated on nerve axon stumps close to the perisynaptic Schwann cells. Scale bar 20 μm. (B) Orthogonal projection of poisoned NMJ showed in (A) to appreciate the axonal localization of the CXCR4 receptor. Scale bar 20 μm. (C) Left panel: Snapshot of the poisoned NMJ showed in the Supporting video, stained with anti‐CXCR4 receptor (red), anti‐neurofilaments (green), merging with Schwann cells expressing GFP (light blue). Scale bar 20 μm. Right panel: Region of interest highlighted by the white square in the left panel, showing that CXCR4 receptor (red) is expressed by the envenomed motor axon terminal (green). Scale bar 5 μm.

Figure S3. NUCC‐390 increases the reformation of motor axon terminals on the soleus muscle after degeneration induced by Bungarus venoms. Representative immunostaining of intoxicated NMJs 96 h after the injection of β‐BTX, *B. caeruleus*, *B. candidus*, or *B. multicinctus* venoms, performed on the same muscles used for the determination of the evoked junction potentials of Figure 2. Left panels: Animals treated with vehicle only; right panels: NUCC‐390‐treated animals. Motor neurons axon terminals are identified by VAMP‐1 immunostaining (green), post‐synaptic nAChRs by fluorescent α‐BTX (red) and the axon by NF staining (white). Scale bars: 50 μm.

Figure 1 Suppvideo. The CXCR4 receptor is expressed on the membrane surface of motor neuron axon. 3D reconstruction of a motor neuron terminal of the soleus muscle taken from a mouse envenomed with *B. caeruleus* venom (quantities as in Figure 1). Schwann cells are visualized owing to transgenic GFP expression in their cytosol and to CXCR4 antibody staining (rabbit monoclonal, Abcam, cat# Ab124824) in red and axonal neurofilaments in green. The morphology of the distribution of red signal indicates that CXCR4 is present on the axonal plasma membrane. The video was generated by 3D viewer plugin of ImageJ software.
