# Supplementary figures and images for: An agonist of the CXCR4 receptor is therapeutic for the neuroparalysis induced by Bungarus snakes envenoming
Source: Clin Transl Med. 2022 Jan 24;12(1):e651. doi: 10.1002/ctm2.651 (PMC8787102; doi:10.1002/ctm2.651)

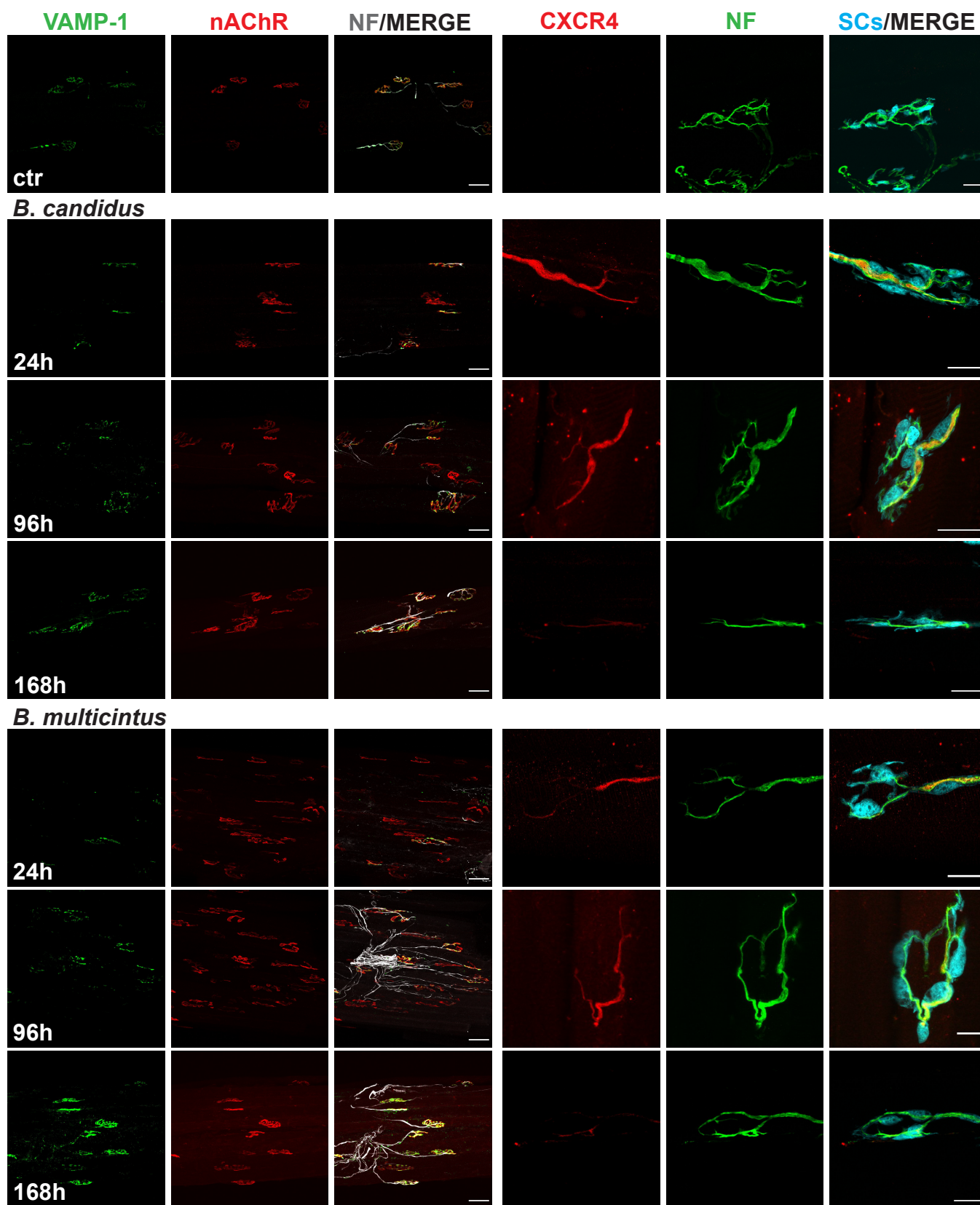

Supplement: Supplementary file 1 — Figure S1. CXCR4 receptor expression on mouse neuronal axons after i.m. injection of Bungarus candidus or Bungarus multicintus venoms in mice. 22 ng/g of B. candidus venom or 22 ng/g of B. multicintus venom was injected in the hind limb of mice, and the soleus muscle was isolated and stained with antibodies specific for the following antigens: first column) VAMP‐1, a marker of motor axon terminals; second column) nicotinic acetyl choline receptor (nAChR), a marker of the post‐synaptic muscle membrane, was stained with fluorescent α‐BTX (α‐BTX, third column) merging with the antibody staining neurofilaments (NF) (chicken polyclonal, Abcam, cat# Ab4680) in grey. The right set of panels displays the staining of samples of soleus muscle, poisoned as above and stained with: first column) anti‐CXCR4 receptor (rabbit monoclonal, Abcam, cat# Ab124824) (red): second column) NF (green); third column) merging with Schwann cells stained in light blue. Symbols of the rows: Samples taken from non‐injected control muscles (ctr); venom treated muscles after 24 h, 96 h and 168 h from i.m. injection of B. candidus or B. multicintus venoms in the hind limb. Notice that the degeneration of the motor axon terminal at the NMJ is complete after 24 h from poisoning as shown by the loss of VAMP‐1 staining. This is accompained by the expression of the CXCR4 receptor on the residual motor axon; this receptor is still present after 4 days from poisoning. A lower magnification was used for the left panels (scale bar 50 μm) to provide a view of several NMJs in the same field, whilst a higher one (scale bar 20 μm) was used in the right panels to better show the expression of the CXCR4 receptor. [file CTM2-12-e651-s003.pdf]

**A**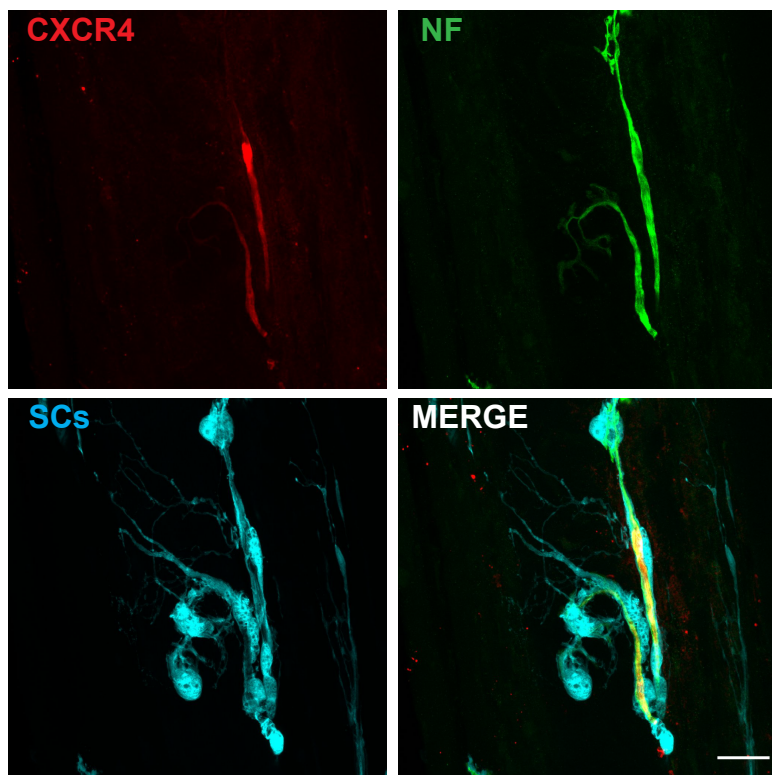**B**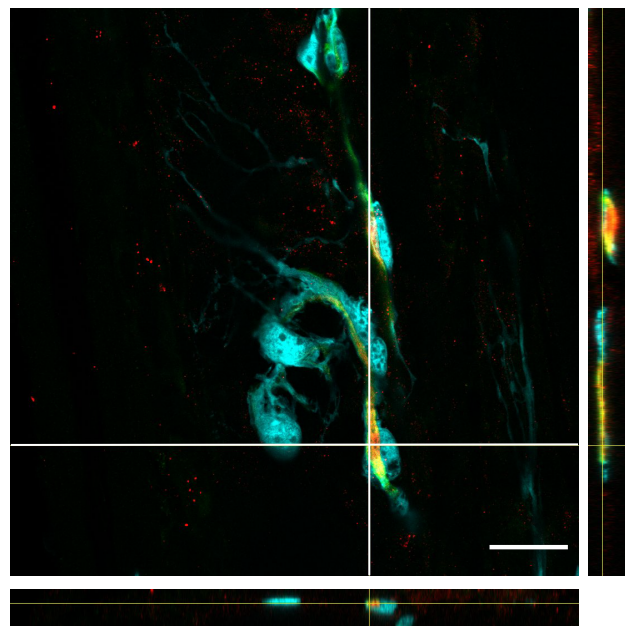**C**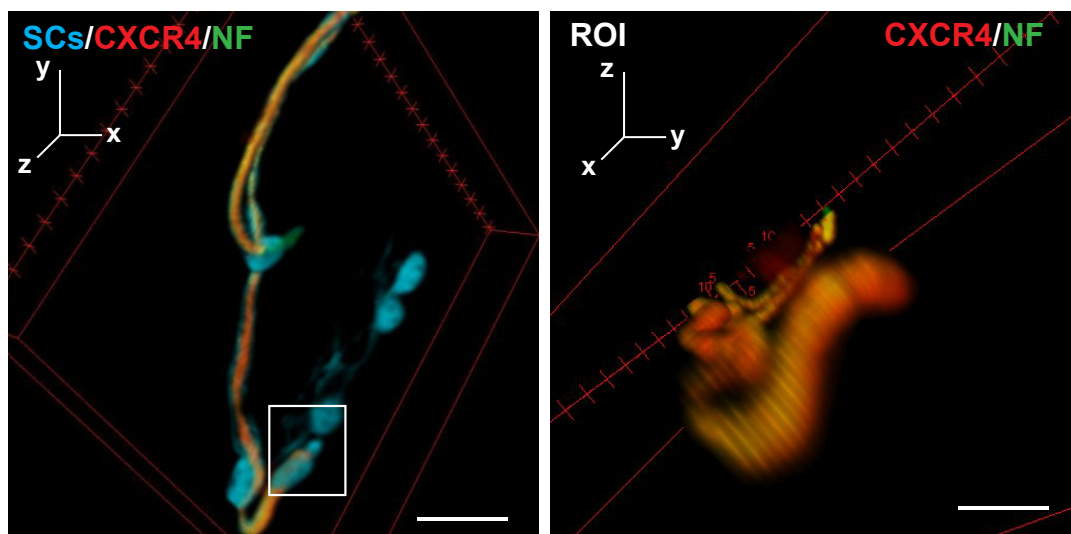

Supplement: Supplementary file 2 — Figure S2. CXCR4 receptor expression on mouse neuronal axons after i.m. injection of Bungarus caeruleus venom. (A) Note that 36 ng/g B. caeruleus venom were injected i.m. in the hind limb of young mice, and the soleus muscle was dissected 96 h after and stained with antibodies specific for the following antigens: anti‐CXCR4 receptor (rabbit monoclonal, Abcam, cat# Ab124824) (red); anti‐neurofilaments (chicken polyclonal, Abcam, cat# Ab4680) in green, merging with Schwann cells expressing GFP (light blue) showing that the CXCR4 staining is concentrated on nerve axon stumps close to the perisynaptic Schwann cells. Scale bar 20 μm. (B) Orthogonal projection of poisoned NMJ showed in (A) to appreciate the axonal localization of the CXCR4 receptor. Scale bar 20 μm. (C) Left panel: Snapshot of the poisoned NMJ showed in the Supporting video, stained with anti‐CXCR4 receptor (red), anti‐neurofilaments (green), merging with Schwann cells expressing GFP (light blue). Scale bar 20 μm. Right panel: Region of interest highlighted by the white square in the left panel, showing that CXCR4 receptor (red) is expressed by the envenomed motor axon terminal (green). Scale bar 5 μm. [file CTM2-12-e651-s004.pdf]

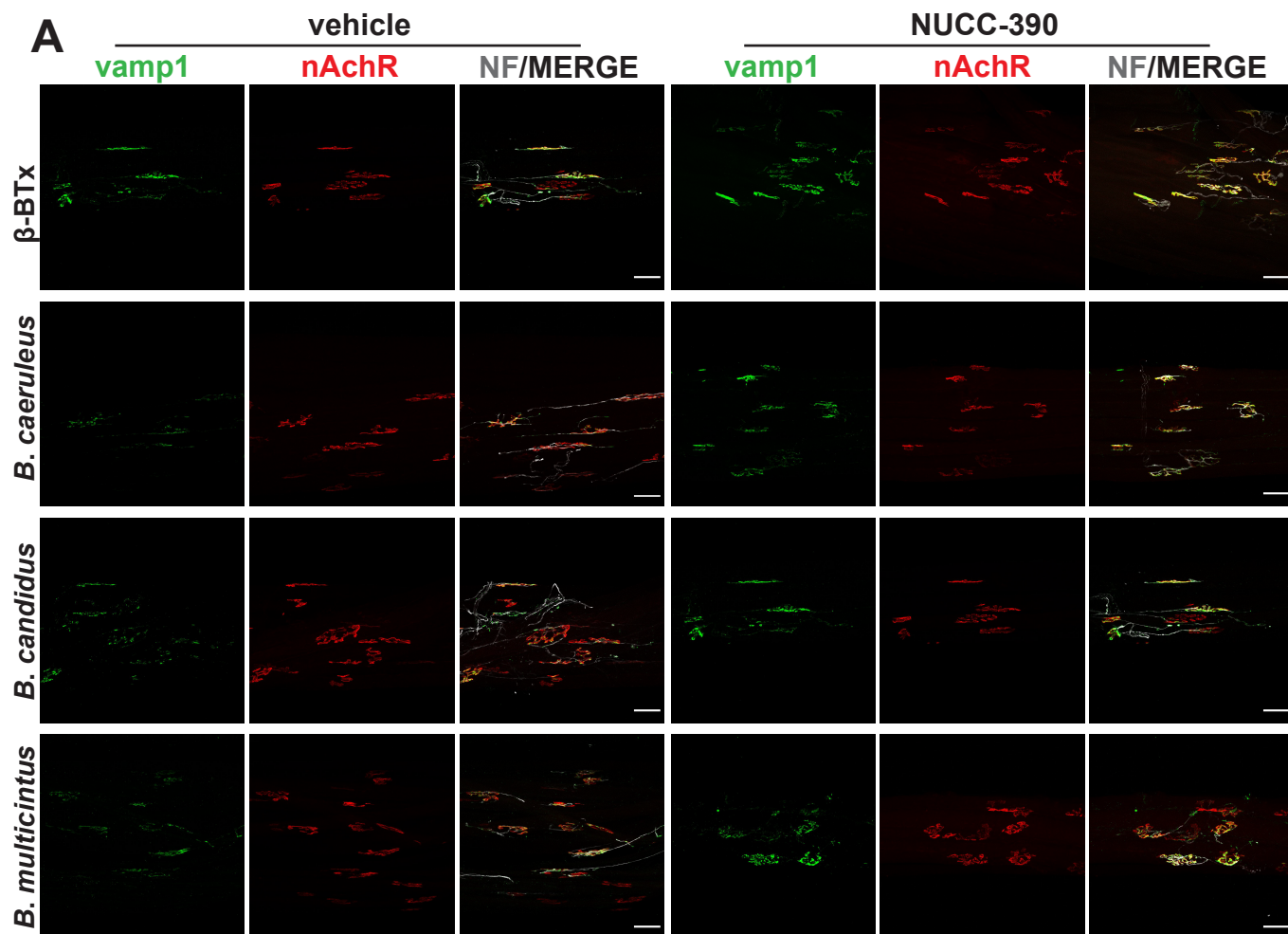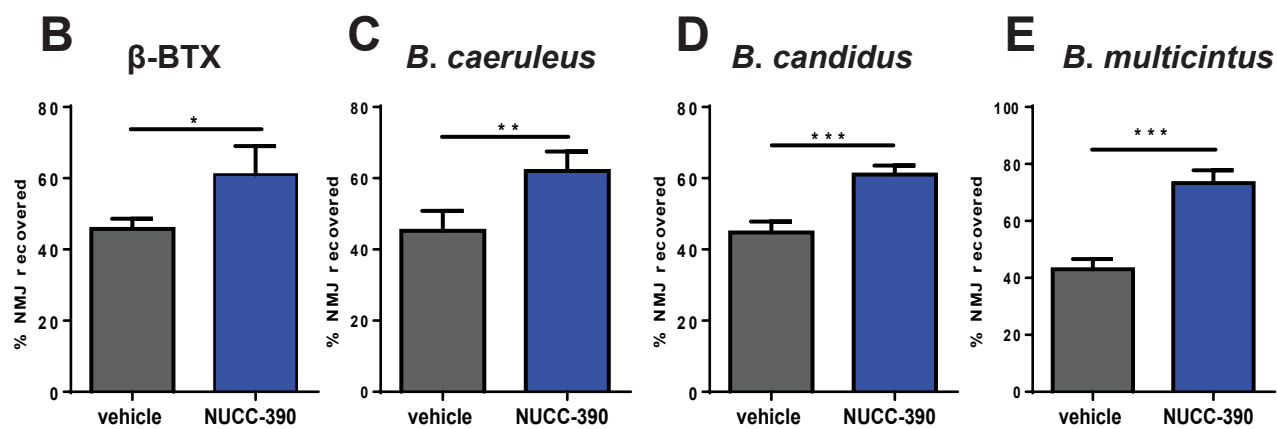

Supplement: Supplementary file 3 — Figure S3. NUCC‐390 increases the reformation of motor axon terminals on the soleus muscle after degeneration induced by Bungarus venoms. Representative immunostaining of intoxicated NMJs 96 h after the injection of β‐BTX, B. caeruleus, B. candidus, or B. multicinctus venoms, performed on the same muscles used for the determination of the evoked junction potentials of Figure 2. Left panels: Animals treated with vehicle only; right panels: NUCC‐390‐treated animals. Motor neurons axon terminals are identified by VAMP‐1 immunostaining (green), post‐synaptic nAChRs by fluorescent α‐BTX (red) and the axon by NF staining (white). Scale bars: 50 μm. [file CTM2-12-e651-s002.pdf]
